# Air Permeability of Maraging Steel Cellular Parts Made by Selective Laser Melting

**DOI:** 10.3390/ma14113118

**Published:** 2021-06-06

**Authors:** Annadurai Dhinakar, Bai-En Li, Yo-Cheng Chang, Kuo-Chi Chiu, Jhewn-Kuang Chen

**Affiliations:** 1 Department of Materials and Mineral Resources Engineering, National Taipei University of Technology, Taipei 106, Taiwan; dhina001mech@gmail.com (A.D.); sssss88043@gmail.com (B.-E.L.); 2 Digital-Can Tech Co., Ltd., Taipei 114, Taiwan; yocheng@digital-can.com (Y.-C.C.); kuochi@digital-can.com (K.-C.C.)

**Keywords:** additive manufacturing, porosity, mold inserts, cellular parts, injection molding, permeability

## Abstract

Additive manufacturing, such as selective laser melting (SLM), can be used to manufacture cellular parts. In this study, cellular coupons of maraging steels are prepared through SLM by varying hatch distance. Air flow and permeability of porous maraging steel blocks are obtained for samples of different thickness based on the Darcy equation. By reducing hatch distance from 0.75 to 0.4 mm, the permeability decreases from 1.664 × 10^−6^ mm^2^ to 0.991 × 10^−6^ mm^2^ for 4 mm thick coupons. In addition, by increasing the thickness from 2 to 8 mm, the permeability increases from 0.741 × 10^−6^ mm^2^ to 1.345 × 10^−6^ mm^2^ at 16.2 J/mm^3^ energy density and 0.14 MPa inlet pressure. Simulation using ANSYS-Fluent is conducted to observe the pressure difference across the porous coupons and is compared with the experimental results. Surface artifacts and the actual morphology of scan lines can cause the simulated permeability to deviate from the experimental values. The measured permeability of maraging steel coupons is regression fit with both energy density and size of samples which provide a design guideline of porous mold inserts for industry applications such as injection molding.

## 1. Introduction

Additive manufacturing (AM) can deliver parts of complex geometries with minimal need for secondary machining. To permit large-scale industrial use [1], cellular structure-based products are prepared using AM in the fields of biomedical [2,3], heat exchanger [4,5] and aeronautical [6] applications.

For injection molding, cooling cycle time is the main factor that affects the throughput [7]. Porous inserts that are permeable to in-mold gas are often used together with cooling channels in steel molds for gas venting [8]. Such porous inserts can be manufactured directly or separately as part of the steel molds via AM which aids in the flexibility of mold designs. Selective laser melting (SLM) has been widely used to additively manufacture metallic parts [9]. It is capable of preparing these porous inserts through controlling the process parameters, including laser power, scan speed, hatch distance, and layer thickness [10].

Energy density in SLM can be represented by the amount of laser power received by the unit volume of materials [11]. The volume undertaking the laser power can be represented by the multiplication of scan speed, hatch distance, and layer thickness. Therefore, the process parameters can lead to varying energy density. By changing energy density, porous structures are formed, and their mechanical properties vary with the degree of porosity [12].

When the porous parts are used for venting purpose in moldings, air permeability is achieved by air flow paths and the topology of pores in cellular structures [13]. Air inside the molding can then escape through the air flow paths in these cellular parts during plastic injection. The amount and pressure of air to escape from the molding depends on the size and shape of the injection molded product. Apparently, the information of air permeability is significant in understanding the release of gas in the molding [14].

There is no theoretical or numerical model which predicts the effect of printing parameter changes on air permeability of SLM porous parts [15]. It is the intention of this study that by varying hatch distance while fixing laser power and scan speed, the cellular parts of different porosity and air permeability are made to correlate with printing parameters. SLM porous parts such prepared can be selected according to their permeability for use in the moldings. Thus, pressurized gas from inside the molding can escape through the pathways within the porous insert to avoid the problems such as short run or burning of porous parts [16].

The current work will provide a guideline for selecting porous parts and their printing parameters based on the permeability needed according to the design of injection parts. Air permeability is obtained according to the Darcy equation by relating pressure drop and flow rate of escaping gas through the porous inserts [17]. In the current study, the cellular parts were prepared using maraging steel by SLM process. Maraging steel has been applied extensively for powder bed fusion AM process and subsequent heat treatment which leads to a combination of high strength and ductility by TRIP effect [18].

We also characterized the cellular structures via computer tomography (CT) to observe the topology of air paths [19]. Furthermore, ANSYS-Fluent is employed to simulate the air flow and to compare the simulated permeability with experimental values. The simulation can be useful in computer-aided-design of porous molds for injection molding and other industrial applications.

## 2. Materials and Methods

### 2.1. SLM of Porous Parts

**Figure 1:**
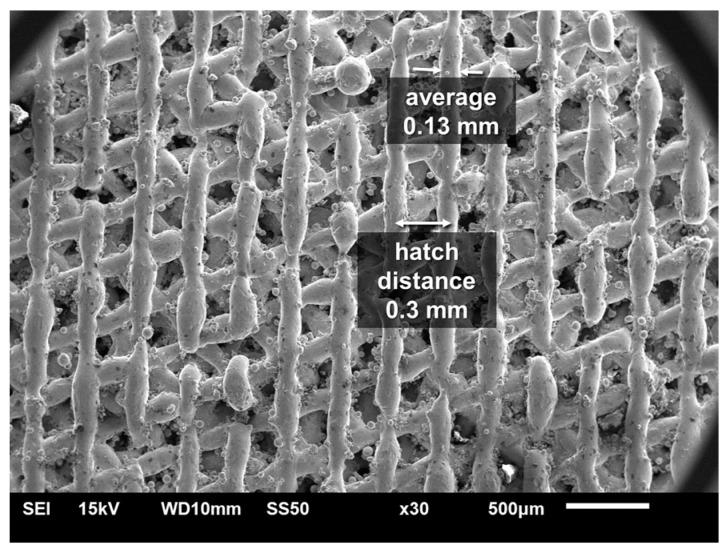
SEM image of printed structures showing average 0.13 mm scan line width and 0.3 mm hatch distance (or D1 specimen in Table 2).

Gas atomized maraging steel powder (MS1, from EOS, Krailling, Germany) of D50 30.7 μm is used in this study and their chemical composition is shown in Table 1. The selective laser melting is made by an EOS M290 (Krailling, Germany) SLM equipment. The energy density is expressed [20] in Equation (1) as:

(1) $$E_{D}\left(J/mm^{3}\right)=\frac{P}{vht}=\frac{285\left(W\right)}{960\left(\mathrm{mm}/s\right)\cdot \;h\left(\mathrm{mm}\right)\cdot \;0.04\left(\mathrm{mm}\right)}$$

where *P* refers to laser power of 285 W, layer thickness (t) is 0.04 mm, and scan speed (v) is 960 mm/s which are fixed in the current study for maraging steel. The hatch distance (h) is varied between 0.3 and 0.75 mm to generate different spacing between laser tracks. The hatch distance is chosen so that a range of porosity can be obtained in the SLM coupons for measurement of air permeability. The energy density is thus controlled in the range of 8.6~21.7 J/mm^3^ by varying hatch distance. Each layer is rotated by an angle of 67° from the previous layer to generate cellular structures as shown in Figure 1.

Nine different energy densities, *E_D_* (J/mm^3^), as listed in Table 2 are used to prepare the porous coupons of (30 × 30 × t) mm. The thickness t ranges from 2 to 8 mm. The thickness is chosen so that air can be permeable through the porous coupons. In the cellular parts of high thickness, limited through pores are available and zero air permeability will not meet the needs for porous molding applications. The porosity of samples is measured directly by comparing the density of samples with the theoretical density of maraging steel being 8.10 g/cm^3^ [21]. Carl Zeiss METROTOM 800 (Oberkochen, GERMANY) is also utilized in this study to make tomography analysis of pore distribution and connectivity in the printed parts [22].

### 2.2. Air Permeability Measurement

**Figure 2:**
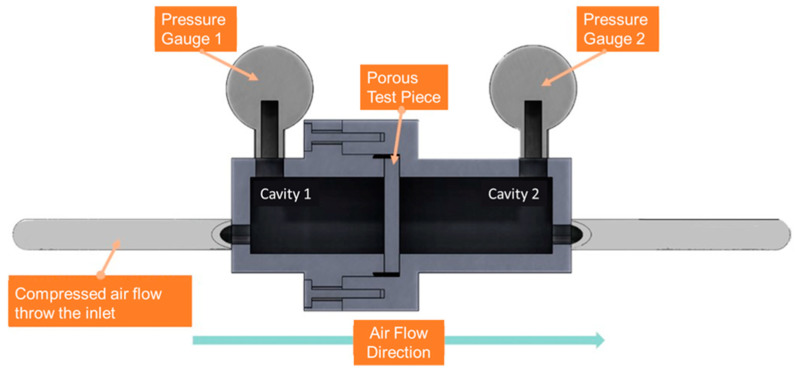
Schematics of the apparatus for air flow across cellular test coupons.

Concept of fluid flow as defined by Darcy equation is used to establish an air permeability measurement device. Air permeability, *k*, is expressed as a relationship between pressure drop (*p_in_*−*p_out_*) and flow rate (*Q*) as Equation (2) [24]:

(2) $$k=\frac{Q\mu L}{A\left(p_{in}-p_{out}\right)}$$

where *L* stands for the thickness of the porous parts, *μ* is the viscosity of air, and *A* is the area for air to pass through. Figure 2 shows the schematic view of air permeability measurement apparatus. The inlet air is supplied by air compressor with pressure regulator. The porous parts are sealed between the inlet and outlet ports with their pressure and flow measured by the gauges and flow sensor. In the experiment, the inlet compressed air pressure is controlled at 0.14, 0.27, or 0.41 MPa to measure the pressure drop and permeability across the cellular coupons made using SLM process parameters listed in Table 2. The cellular SLM specimens are mounted so that the area of 30 × 30 mm^2^ is available for air to flow through. The experiment is also conducted by varying the thickness of the test coupons to observe the change of permeability with sample size.

### 2.3. Numerical Simulation and Analysis

**Figure 3:**
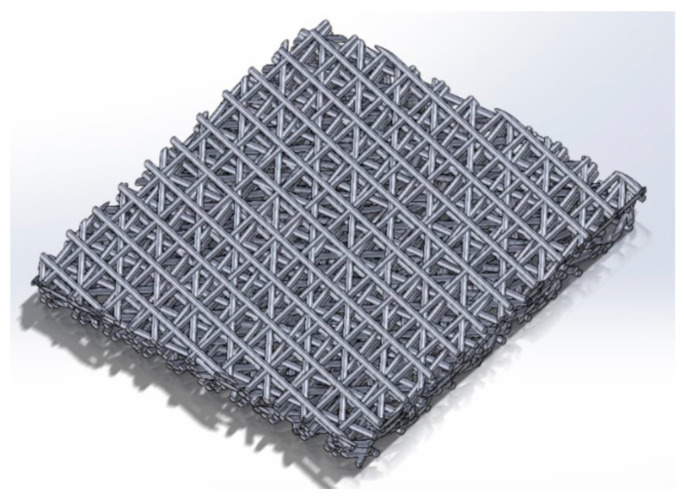
CAD sample constructed using Solidworks for D5 sample.

CAD (Computer-Aided Design) structure is developed using Solidworks software for ANSYS-Fluent (v.2019 R3, Canonsburg, PA, USA) simulation. In Solidworks software, circular cylinders of diameter 0.13 mm are employed as the scanning line width as shown in Figure 3. Each layer is rotated by an angle of 67° from the previous layer to generate porous gaps between the layers. The samples are square of 30 × 30 mm^2^ and their thickness varying from 2 to 8 mm to replicate the experimental samples.

**Figure 4:**
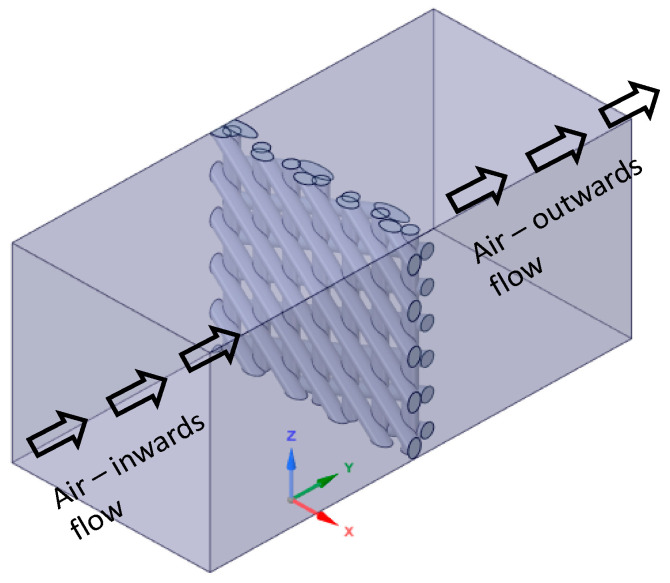
Compressed air flow through cellular coupons shown for D3 sample.

**Figure 5:**
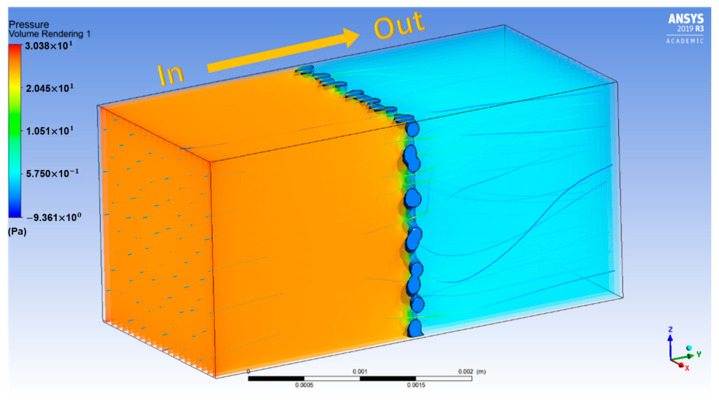
Schematic view of flow field in simulation showing the pressure drop (left: gas inlet and right: gas outlet).

In ANSYS-Fluent, compressed air flow is modelled to pass through the cellular coupons as illustrated by Figure 4 in a closed enclosure. Square walls in the closed envelope are named after inlet and outlet flow, whereas the rectangular walls that surround the flow region are named as stationary walls. The pressure and velocity of the flow are controlled for the inlet wall with the same parameters employed in the experiment. The stationary walls of the envelope hold no-slip condition. Virtual laminar air flow is performed to simulate and validate experimental results [24]. Based on the mesh convergence study, a mesh size of around 0.05 mm is optimal for description of the complex geometries of the sample. Global mesh size of 0.05 mm for the 4-node tetrahedral element is used to mesh the CAD specimens [25]. Exemplary simulation result is shown in Figure 5 demonstrating the air pressure drop across the cellular coupon.

Darcy equation is employed to calculate permeability using the pressure drop simulated across the cellular coupons. Permeability results from the numerical simulation are then obtained and compared with the experiment results for all design variations.

## 3. Results and Discussion

### 3.1. Effects of Energy Density and Thickness on Sample Porosity

**Figure 6:**
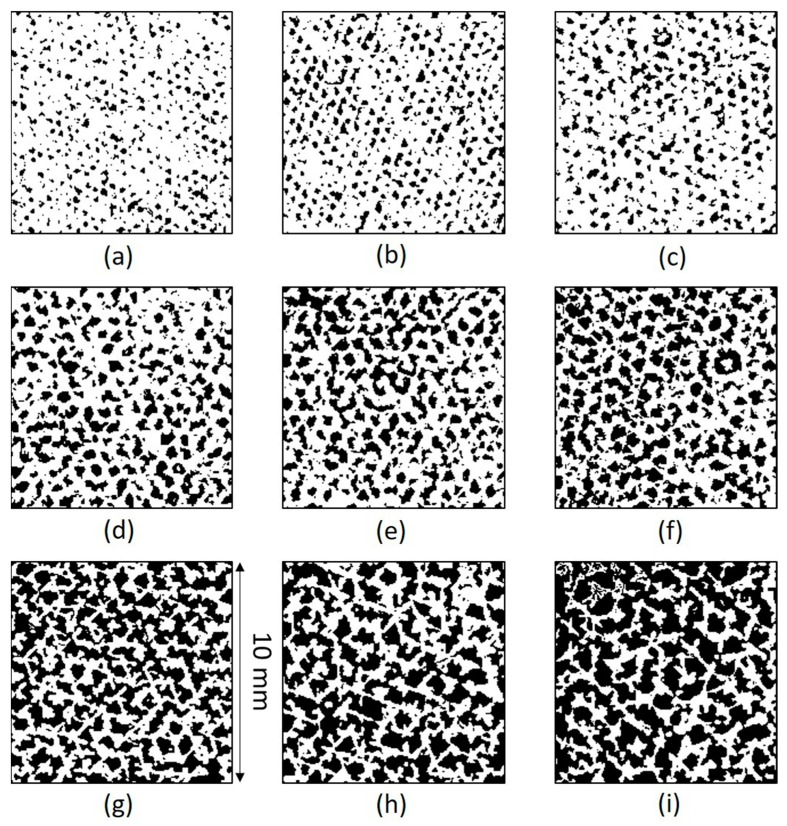
Binary image showing projected pores (black parts) by surface grinding the (**a**) D1, (**b**) D2, (**c**) D3, (**d**) D4, (**e**) D5, (**f**) D6, (**g**) D7, (**h**) D8, and (**i**) D9 samples, respectively. (The width and length of each image corresponds to the surface area of 10 mm × 10 mm).

**Figure 7:**
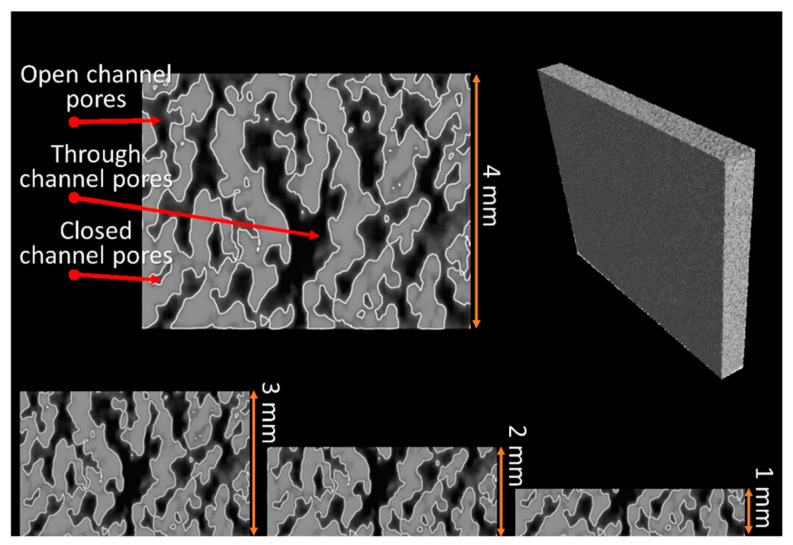
CT images of pores changing with thickness of samples. Vertical direction corresponds to the thickness direction of the samples. The inset of 1~3 mm represents the thickness of the samples.

**Figure 8:**
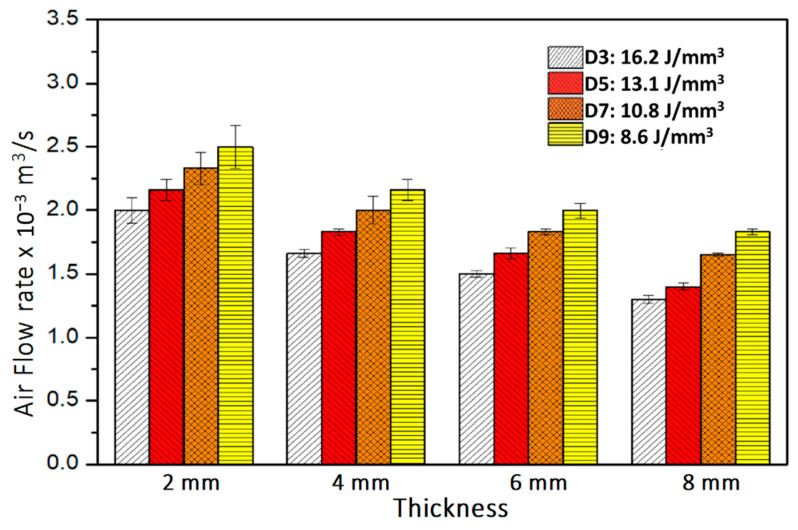
Flow rate of air through porous coupons for variation in thickness and energy density when inlet pressure is 0.27 MPa.

Porosity of the cellular coupons is evaluated from sample density and is shown in Table 2 to vary with energy density. Figure 6 shows the binary images of the projected porous areas in black. The area fractions of pores (Table 2) are slightly larger than the volumetric porosity due to the projected porous area being a two-dimensional section of the three-dimensional porous object. The 2D projected porosity and the actual porosity obtained from density measurement have a linear relation with 0.94 R^2^ (coefficient of determination). Porosity apparently increases from 21.5% to 49.5% with energy density decreasing from 21.7 to 8.6 J/mm^3^. The structural morphologies of air pathways can be observed in the reconstructed CT images as shown in Figure 7. Through channels are channels that are open to both the top and bottom surfaces. Open channels represent the channels that are open on one surface only, whereas closed channels correspond to closed pore capsules between metals. Among the different types of channels, only through channels contribute to the air flow. When the thickness of the coupon decreases, some close pores become open channels, and some open channel pores become through channels. The flow rate of compressed air through the cellular coupons is shown in Figure 8. Lower thickness and energy density results in greater numbers of through channels which lead to greater air flow and higher air permeability. Therefore, energy density can be tuned to tailor the porosity and permeability of cellular parts [26].

### 3.2. Effects of Porosity and Inlet Air Pressure on Air Permeability

**Figure 9:**
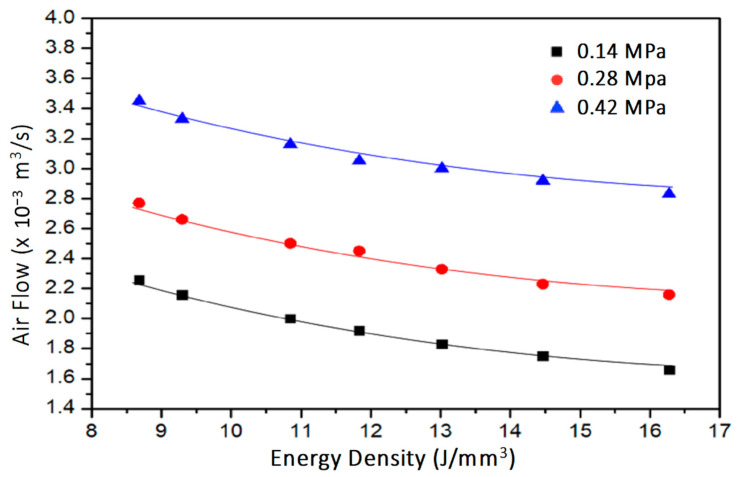
Change of air flow rate across the 4 mm cellular coupons made by different energy densities and tested at three inlet pressures.

**Figure 10:**
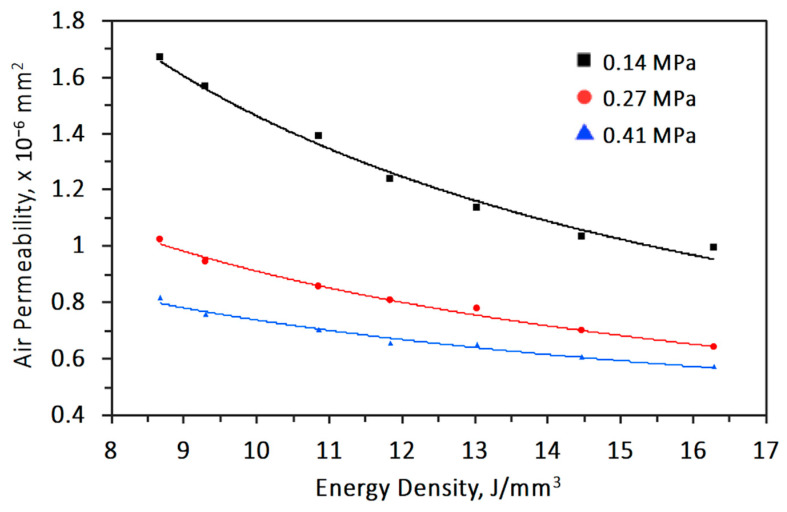
Relationship between air permeability and energy density for 4 mm thickness coupons tested at different inlet pressures.

At fixed inlet pressure and cellular coupon thickness, air permeability is calculated from the pressure drop, flow rate, and air viscosity according to Equation (2). Figure 9 shows the air flow rate of 4 mm thick cellular coupons made by varying energy density and inlet air pressure. An increase of energy density and decrease in inlet air pressure decreases the air flow. Air permeability is deducted and shown in Figure 10. The air permeability obviously increases from 0.99 × 10^−6^ mm^2^ to 1.664 × 10^−6^ mm^2^ for decreasing energy density from 21.7 J/mm^3^ to 8.6 J/mm^3^ at inlet air pressure of 0.27 MPa. The increase of air permeability is related to the increased porosity due to decreasing energy density, or there are more through channels (Figure 7) as air pathways in the samples.

**Figure 11:**
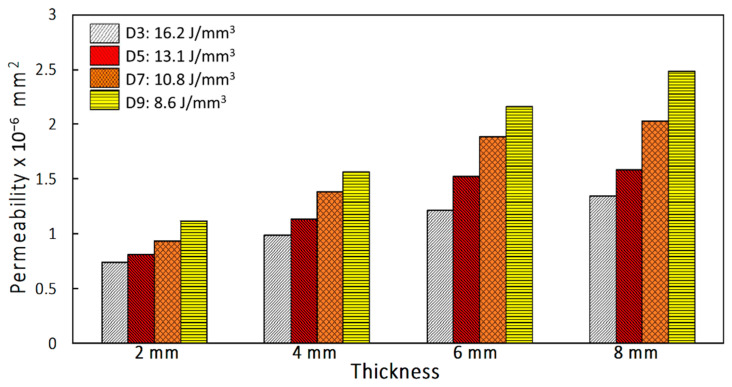
Permeability as a function of energy density (J/mm^3^) and coupon thickness at inlet pressure of 0.14 MPa.

Furthermore, at any specific energy density, permeability drops with increasing inlet pressure. With increasing inlet pressure, the air flow rate must also increase. When the pressure drop increases at a higher rate than the increase of flow rate, air permeability drops. Figure 11 shows that by increasing thickness of coupons, air permeability also increases. For the thickness varying from 2 to 8 mm and energy density varying from 16.2 to 8.6 J/mm^3^, the air permeability increases from 0.741 × 10^−6^ to 2.485 × 10^−6^ mm^2^ when inlet pressure is 0.14 MPa. With these measurements, it is possible to reproduce the morphological and geometrical of cellular coupons and vary the air permeability in a controlled manner [27].

### 3.3. Simulation for Air Permeability

**Figure 12:**
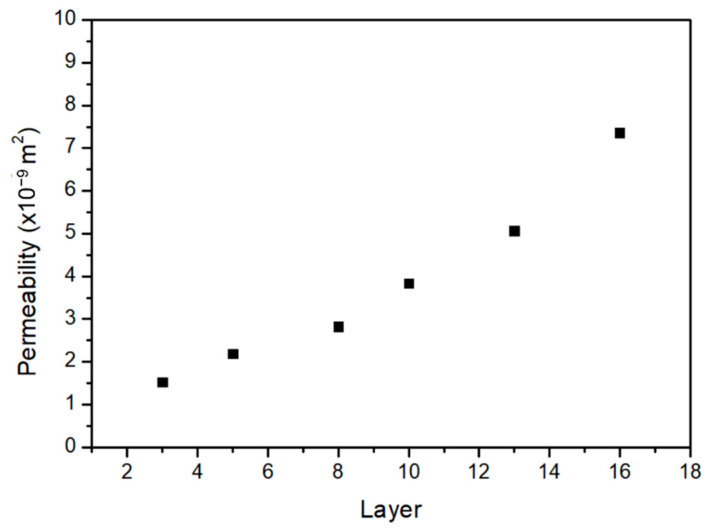
Relationship between permeability with number of layers obtain through simulation at inlet pressure 0.14 MPa.

**Figure 13:**
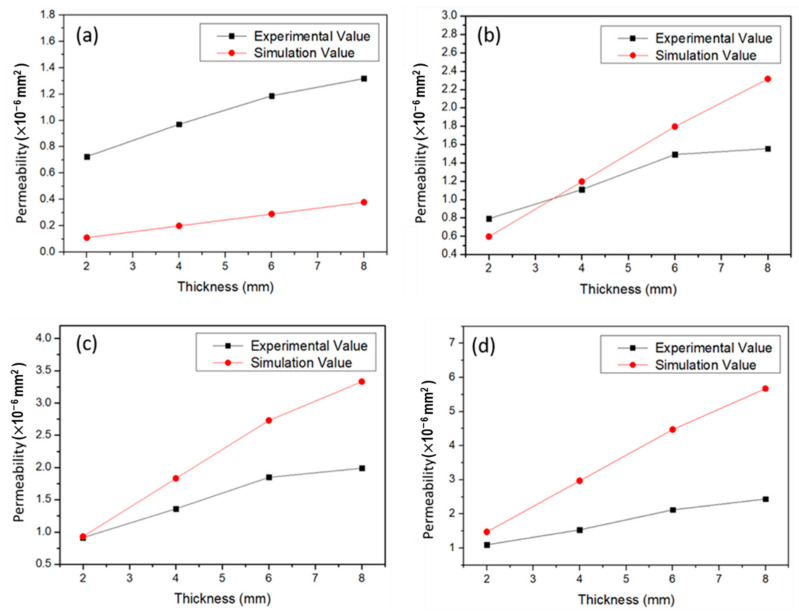
Comparison between permeability of cellular coupons obtained using experimental and simulation for (**a**) D3, (**b**) D5, (**c**) D7 and (**d**) D9 conditions at 0.14 MPa inlet pressure.

Pressure drop across the cellular coupons is simulated to calculate the permeability. The air permeability obtained through ANSYS-Fluent simulation increases with the number of layers as shown in Figure 12 which has the same trend as that in Figure 11. In Figure 13a–d, permeability measured through experiment and simulation are compared for different energy densities. For samples of 2 mm thickness made at 13.1 J/mm^3^ energy density, simulation accurately fits with the experimental results. At low energy density (8.6 J/mm^3^ of Figure 13d), simulated permeability is somewhat higher than the measured value. While at higher energy density, such as 16.2 J/mm^3^ (Figure 13a), permeability is lower than the experimental values. This result demonstrates that the permeability is also affected by the geometrical variation of the cellular structures due to manufacturing process.

For cellular parts printed using lower energy density, struts are often partially sintered. Struts of next layer stack on the unsintered scan lines and effectively reduce the layer thickness. Therefore, the cross sections of the struts printed through the SLM process are elliptical in shape [28] with shorter diameter along the printing direction. These are in contrast to the round cylindrical shapes of the designed CAD used in simulation. The measured permeability is thus lower than that of simulated cellular structures.

On the other hand, the simulation underestimates the permeability for samples printed with high energy density. It is mainly due to the fact that the simulated strut diameter used the actual average strut diameter based on observations. The surfaces of struts are attached with many partially melted particles. The simulated strut diameter is thus larger in diameter than the actual strut diameters with surface roughness [29]. The scan lines with larger round cylindrical sections lead to lower permeability in the simulations of parts printed with high energy density. Therefore, it is important to bear the effects of process parameters on surface morphology [30] in mind for optimum CAD modelling.

### 3.4. Empirical Expression of Permeability

**Figure 14:**
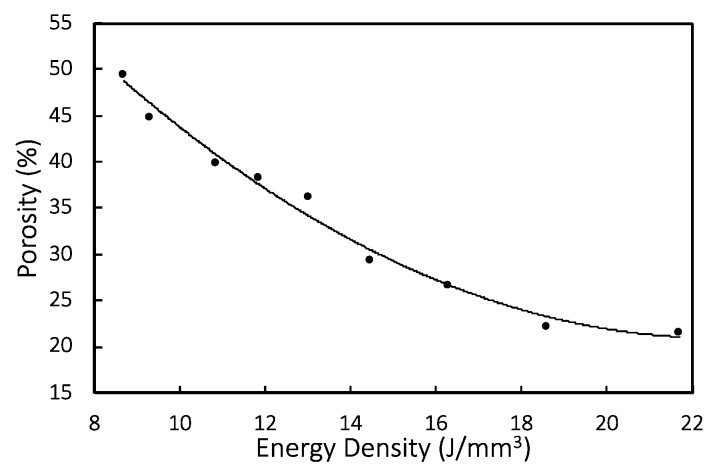
Relationship between experimental energy density and porosity shown with regression equation.

Figure 14 shows the relationship between energy density and porosity. The porosity from experiments drops with increasing energy density used for SLM process and can be correlated exponentially with a coefficient of determination R^2^ being 0.96:

(3) $$Porosity\left(\%\right)=84.32\times e^{-0.068ED}$$

where *ED* is the energy density in J/mm^3^.

According to Hommel et al. [31], several models have been used to express the change of permeability with porosity. Among them, power law is one that is often used to relate porosity and permeability. Parameters such as energy density and sample thickness could be taken into consideration to relate with air flow. Besides porosity in the samples, decreasing samples thickness and increasing inlet pressure both increases air permeability (Figure 10 and Figure 11) as well. The air permeability regression equation relating experimental permeability, energy density (*ED* in J/mm^3^), inlet air pressure (*p* in MPa), and sample thickness (*t* in mm) is thus fitted empirically by Equation (4) with a 0.97 R^2^.

(4) $$k\;\left(m^{2}\right)=3.4488\times 10^{-13}\;e^{-0.0622\;ED}p^{-0.621}t^{0.582}$$

**Figure 15:**
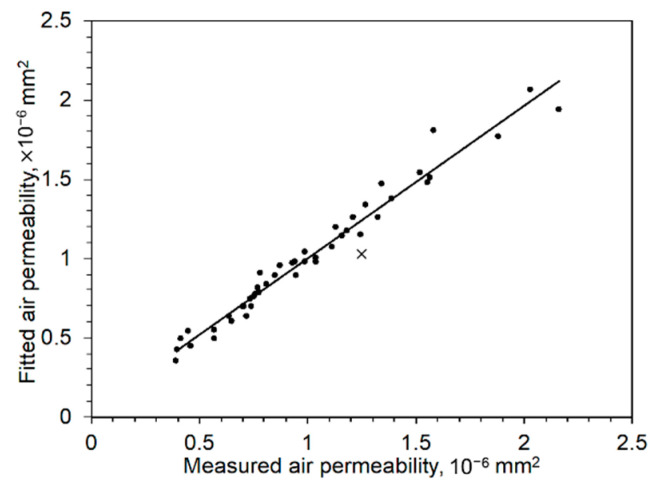
Comparison between fitted permeability and experimental permeability.

This empirical equation is useful when designing the porous inserts for injection molding. Figure 15 compares fitted and experimental results. The fitted values based on Equation (4) matches well with the experiment and follows the Darcy regime of viscous flow [32]. According to Chan et al. [33], permeability routinely used in the paper industry is often discussed via the Kozeny–Carman equation using a simple power law model. Sabet uses this model to bring a relationship between permeability and Knudsen number [34] which refers to the ratio of pore size divided by sample thickness. Even though Knudsen number speaks about the capability of mean molecular flow, the combined effect of pressure and thickness on air flow also demonstrates similar behaviors.

## 4. Conclusions

Process parameters of selective laser melting are varied to control the energy density and to print cellular coupons of varying porosity. Air permeability is measured for maraging cellular parts made using different hatch spacing, sample thickness at three air flow pressure. Air permeability makes a negative exponential relationship to the energy density. When energy density exceeds 18.6 J/mm^3^ and porosity becomes lower than 22.3%, the air permeability approaches zero due to the lack of through holes in the cellular parts of 4 mm thickness. Air permeability is shown to be inversely proportional to inlet pressure to the power of 0.621 and directly proportional to thickness to the power of 0.582. The relations of energy density, porosity, thickness, and air permeability have been expressed in empirical equations (3) and (4). The fitted values relate well with the experimental values. These regression results will be valuable for engineering purposes in the design of AM porous molding for injection molding applications.

ANSYS-Fluent simulation is helpful in understanding the characteristics of cellular structure and air permeability. For the 2 mm thickness and 13.1 J/mm^3^ energy density, simulation results match well with the experiment. However, the simulation cannot account for the irregular surface roughness and morphology of the struts. Therefore, the discrepancies between the simulated structures and the geometry features on actual SLM samples will be taken into account for more accurate simulations.

## Figures and Tables

**Table 1:** Composition percentages of maraging steel powder for SLM [23].

C	Ni	Co	Mo	Ti	Al	Mn	Si	Fe
<0.03	17–19	8.5–9.5	4.2–5.2	0.6–0.8	0.05–0.15	<0.1	<0.1	balance

**Table 2:** SLM process parameters and porosity as obtained with fixed 285 W laser power, 960 mm/s scanning speed, and 40 µm layer thickness. D1~D9 identify the samples made by changing hatching distances.

Sample	D1	D2	D3	D4	D5	D6	D7	D8	D9
Hatch distance (mm)	0.3	0.35	0.4	0.45	0.5	0.55	0.6	0.7	0.75
Energy density (J/mm^3^)	21.7	18.6	16.2	14.4	13.1	11.8	10.8	9.3	8.6
Porosity (%)	21.5	22.3	26.6	29.3	36.2	38.3	39.9	44.9	49.5
Projected area fraction of pores (%)	25.9	28.6	38.1	41.5	45.4	49.6	54.2	56.5	57.1

## Data Availability

Data sharing is not applicable.
